# Retraction: Colonic Gallstone Ileus: A Rare Etiology of Large Bowel Obstruction

**DOI:** 10.7759/cureus.r113

**Published:** 2024-01-25

**Authors:** Abdulaziz O Alshehri, Turki S Aljuhani, Salihah S Alotaibi, Shahad A Almughamisi, Mariam M Ageel, Abdulmuhsen H Alameer, Khalid M Alqahtani, Ziyad A Alhumaid, Abdullah S Alsuwayeh, Mohammmad S Almarri, Saja F Almotadaris, Hamad Y Alsaeed, Abdallh M Alatwai, Ahmed M Alatawi, Faisal Al-Hawaj

**Affiliations:** 1 College of Medicine, University of Debrecen, Debrecen, HUN; 2 College of Medicine, University of Hail, Hail, SAU; 3 College of Medicine, AlMaarefa University, Ad Diriyah, SAU; 4 College of Medicine, King Abdulaziz University, Jeddah, SAU; 5 General Practice, Al-Ahsa Health Cluster, Al-Ahsa, SAU; 6 College of Medicine, King Khalid University, Abha, SAU; 7 College of Medicine, King Saud bin Abdulaziz University for Health Sciences, Riyadh, SAU; 8 College of Medicine, Imam Mohammed Ibn Saud Islamic University, Riyadh, SAU; 9 General Surgery, Adan Hospital, Hadiya, KWT; 10 Unaizah College of Medicine, Qassim University, Qassim, SAU; 11 College of Medicine, Jordan University of Science and Technology, Irbid, SAU; 12 College of Medicine, Tabuk University, Tabuk, SAU; 13 College of Medicine, Imam Abdulrahman Bin Faisal University, Dammam, SAU

The Editors-in-Chief have retracted this article. Concerns were raised regarding the identity of the authors on this article. Specifically, Faisal Alhaway and Malak Shammari have stated that they were added to this article without their knowledge or approval. The identity of the other authors could also not be verified. As the appropriate authorship of this work cannot be established, the Editors-in-Chief no longer have confidence in the results and conclusions of this article.

